# Identification of Potential Citrate Metabolism Pathways in *Carnobacterium maltaromaticum*

**DOI:** 10.3390/microorganisms9102169

**Published:** 2021-10-18

**Authors:** Heng Li, Nancy E. Ramia, Frédéric Borges, Anne-Marie Revol-Junelles, Finn Kvist Vogensen, Jørgen J. Leisner

**Affiliations:** 1 Pasteurien College, Soochow University, Suzhou 215123, China; hli@suda.edu.cn; 2 Department of Veterinary and Animal Sciences, Faculty of Health and Medical Sciences, University of Copenhagen, DK-1870 Frederiksberg, Denmark; 3 Laboratoire d’Ingénierie des Biomolécules (LIBio), Ecole Nationale Supérieure d’Agronomie et des Industries Alimentaires (ENSAIA), Université de Lorraine, LIBio, F-54000 Nancy, France; ramia.nancy@gmail.com (N.E.R.); frederic.borges@univ-lorraine.fr (F.B.); anne-marie.revol@uni-lorraine.fr (A.-M.R.-J.); 4 Department of Food Science, Faculty of Science, University of Copenhagen, DK-1958 Frederiksberg, Denmark; fkv@food.ku.dk

**Keywords:** lactic acid bacteria, cheese, citric acid, oxaloacetate, diacetyl

## Abstract

In the present study, we describe the identification of potential citrate metabolism pathways for the lactic acid bacterium (LAB) *Carnobacterium maltaromaticum*. A phenotypic assay indicated that four of six *C. maltaromaticum* strains showed weak (Cm 6-1 and ATCC 35586) or even delayed (Cm 3-1 and Cm 5-1) citrate utilization activity. The remaining two strains, Cm 4-1 and Cm 1-2 gave negative results. Additional analysis showed no or very limited utilization of citrate in media containing 1% glucose and 22 or 30 mM citrate and inoculated with Cm 6-1 or ATCC 35586. Two potential pathways of citrate metabolism were identified by bioinformatics analyses in *C. maltaromaticum* including either oxaloacetate (pathway 1) or tricarboxylic compounds such as isocitrate and α-ketoglutarate (pathway 2) as intermediates. Genes encoding pathway 1 were present in two out of six strains while pathway 2 included genes present in all six strains. The two potential citrate metabolism pathways in *C. maltaromaticum* may potentially affect the sensory profiles of milk and soft cheeses subjected to growth with this species.

## 1. Introduction

The main metabolic activity of lactic acid bacteria (LAB) in milk fermentation is lactic acid production. Various species and strains of LAB, including *Carnobacterium maltaromaticum* isolated from Mozzarella cheese, are also able to metabolize citrate, which leads to the production of aroma compounds [1,2,3,4] *. C. maltaromaticum* is common in milk and soft cheeses as well as in meat and fish products. It is also isolated from a range of cold and temperate environments. Some strains of *C. maltaromaticum* (previously named *Carnobacterium piscicola)* are fish pathogens [5,6,7,8].

The presence of *C. maltaromaticum* in milk and soft cheeses may affect the sensory profiles of such products [9]. In this context, citrate is of interest as LAB produces flavor compounds from this compound, including acetate, diacetyl, acetoin, and 2,3-butanediol [1,3,4,10,11,12].

To extend our knowledge on potential citrate metabolism pathways in *C. maltaromaticum*, we here compare phenotypic tests for citrate metabolism with analysis of whole-genome sequences to identify genes predicted to be involved in citrate metabolism. The main aim was to supply information on the potential sensory applicability of using *C. maltaromaticum* as adjunct cultures in soft cheeses.

## 2. Materials and Methods

### 2.1. Strains Collection, Phenotypic Tests, and Bioinformatic Analyses

Table 1 lists *C. maltaromaticum* strains used in phenotypic tests and bioinformatics and their sources.

### 2.2. Phenotypic Tests for Citrate Metabolism and Test for Growth on a Citrate-Containing Medium

An agar medium for the detection of citrate utilizing LAB was modified from the procedure described by Kempler and McKay [14]. The basal medium contained 0.25% milk protein hydrolysate peptone (Bacto, Mt Pritchard, Australia) and 1.5% agar, autoclaved at 121 °C for 15 min, after which sterile 0.4% nonfat milk (Arla Foods, Aarhus, Denmark) and 0.5% w/v glucose were added, and pH was adjusted to 6.6. Finally, two solutions, one containing 10% potassium ferricyanide and one containing 1 g ferric citrate and 1 g sodium citrate in 40 mL water, were heat-treated at 100 °C for 30 min and 10 mL of each solution was added to 1 L agar medium. Plates were dried in the dark overnight at 30 °C and bacterial cultures were streaked, either directly from frozen culture vials or an overnight culture propagated in All Purpose Tween broth (APT) (Difco, Sparks, MD, USA). Incubation was performed under aerobic or microaerophilic conditions (plates were incubated in a jar with air exhausted by a tea light) in the dark at 25 °C. Cultures were scored positive if they developed a blue color after 3 or 5 days.

A determination of citrate metabolism by chemical analysis was carried out by sub-culturing the Cm 6-1 and ATCC 35586 strains in APT broth (Difco, Beckton, Dickinson and Company, Sparks, MD, USA) with 1% glucose and 22 or 30 mM citrate. Strains were sub-cultured twice o/n in APT broth at 25 °C before adding 10 µl of 22 h cultures to 10 mL of APT broth. The cultures were incubated anaerobically (Anaerogen, Oxoid, Basingstoke, UK) for 9 days at 25 °C before sampling. Viable counts were determined by the transfer of appropriate dilutions of sterile peptone saline to APT agar plates that were incubated for three days anaerobically at 25 °C before enumeration. Citric acid was determined by MS-Omics (Vedbæk, Denmark) using a gas chromatography-mass spectrometry (GC-MS) method. Samples were derivatized as described by Smart et al. [15] and the derivatization reaction was stopped by the addition of chloroform. Subsequently, the chloroform phase with derivatives was injected into the GC. All samples were analyzed in a randomized order. Analysis was performed using gas chromatography (7890B, Agilent, Santa Clara, CA, USA) coupled with a quadrupole mass spectrometry detector (5977B, Agilent). The system was controlled by ChemStation (Agilent). Raw data were converted to netCDF format using Chemstation. Then, the data were imported and processed in MATLAB R2018b (Mathworks, Inc., Natick, MA, USA) using the PARADISe software described by Johnsen et al. [16].

Sample analysis for contents of short-chain fatty acids was carried out by MS-Omics as follows. Samples were acidified using hydrochloride acid, and deuterium-labeled internal standards were added. All samples were analyzed in a randomized order. Analysis was performed using a high polarity column (Zebron™ ZB-FFAP, GC Cap. Column 30 m × 0.25 mm × 0.25 µm) installed in a GC coupled with a quadropole detector and controlled by ChemStation, similar to the analysis for citric acid. Raw data was converted to netCDF format using Chemstation before the data was imported and processed in Matlab R2014b (Mathworks, Inc.) using the PARADISe software [16].

### 2.3. Whole-Genome Sequencing

Isolates were grown on APT agar for 24 h at 30 °C and genomic DNA was extracted and purified using the DNeasy Blood & Tissue Kit, according to the manufacturer’s instructions (Qiagen GmbH, Hilden, Germany). The quality of the extracted DNA was assessed using a Nanodrop ND-1000 spectrophotometer (Nanodrop Technologies, Wilmington, DE, USA) and electrophoresis on a 1.0% (*w*/*v*) agarose gel. Sequencing was performed using MiSeq (Illumina) at a 300 bp paired-end read format. Sequencing reads were de novo assembled with CLC Genomics Workbench 10.1.1 (Qiagen, Aarhus, Denmark).

### 2.4. Bioinformatic Analyses for Genome Annotation

The presence of genes included in citrate metabolism pathways 1 and 2 were examined for a number of *Carnobacterium* spp. whole-genome sequences. A single nucleotide polymorphism (SNP) cladogram was constructed using *Carnobacterium* spp. sequences downloaded from NCBI GenBank (n = 21), RAST annotation server (n = 1), and the *C. maltaromaticum* isolates from the present study (n = 6). The annotations of the pathways were predicted via the RAST annotation server [17,18,19]. The SNP tree was constructed by CSI Phylogeny 1.4 (https://cge.cbs.dtu.dk, accessed on 17 June 2021) with ATCC 35586 as the reference genome. The select minimum depth at SNP positions was set at 10x. The select minimum relative depth at SNP positions was 10%. The select minimum distance between SNPs (prune) was 10 bp, while the select minimum SNP quality was 30. Finally, the select minimum read mapping quality was 25 and the select minimum Z-score was set at 1.96. Then, the phylogenetic tree was imported to iTOL (Interactive Tree of Life) for visualization (https://itol.embl.de, accessed on 17 June 2021) [20]. Leaf sorting was set as the default and the tree was re-rooted at midpoint with ATCC 35586 as the reference genome. 

Detailed genome annotations were carried out using a combined bioinformatical approach including the RAST annotation server [17,18,19], SMART [21], Pfam 32.0 [22], Uniprot database [23], KEGG database [24], and local Blast within the CLC Genomics Workbench 10.1.1. Briefly, whole-genome assemblies were uploaded to the seed-based RAST annotation server for genome annotation and in MicroScope [25]. To improve the annotation accuracy for genes found to be associated with citrate metabolism, based on the KEGG pathway website, sequence alignments for specific genes were downloaded from the Pfam and Uniprot databases. The hits with an e-score of 1e^−20^ or lower were considered as indicative of putative functional proteins or enzymes [6]. 

**Figure 1:**
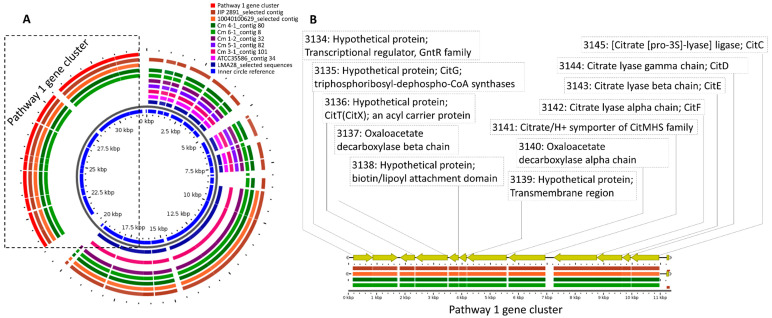
(**A**) Comparison of strains with contigs including genes encoding pathway 1. For comparison, whole-genome sequences of three strains included in the initial Single Nucleotide Polymorphism analysis (SNP) (Figure S1) were added: JIP 2891 and 10040100629 are sequences for isolates from an adult, diseased pike, and LMA28 is a sequence of an isolate from soft, ripened cheese. (**B**) Example of the genes predicted to be included in pathway 1 in the *C.* *maltaromaticum* Cm 6-1 strain.

**Figure 2:**
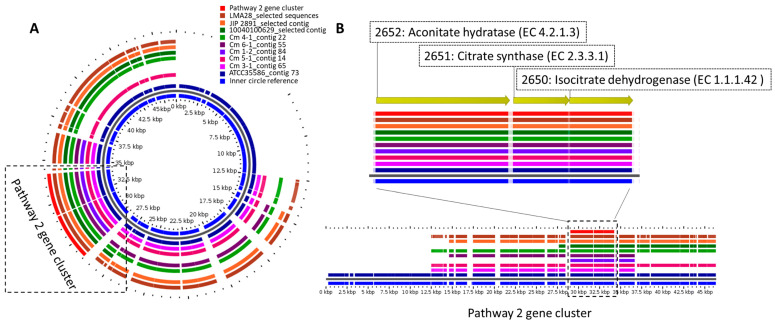
(**A**) Comparison of strains with contigs including genes encoding pathway 2. Three additional strains were included for comparison, see legend to Figure 1A. (**B**) Example of the genes predicted to be included in pathway 2 in the *C.* *maltaromaticum* Cm 6-1 strain.

Moreover, the contigs containing the *cit* operon were retrieved from the whole-genome sequence and blasted against all the strains. The results were visualized in GView (Figure 1 and Figure 2 ) [26]. The presence of transmembrane helices was predicted by using the TMHMM server [27] at DTU Bioinformatics at the Department of Bio and Health Informatics of the Technical University of Denmark. Protein domain predictions were obtained from SMART (http://smart.embl-heidelberg.de, accessed on 17 June 2021). Figure 1 and Figure 2 list the annotated gene products encoding the functions of citrate uptake or citrate metabolism with oxaloacetate as an intermediate (pathway 1) or the truncated TCA cycle (pathway 2).

## 3. Results and Discussion

*C. maltaromaticum* citrate metabolism was tested on a citrate-containing agar medium [2,14]. The positive controls, *Enterococcus faecalis* ATCC 29212, *Lactiplantibacillus pentosus* DSMZ 20314, and *Lactiplantibacillus plantarum* LMG 11405, all showed strong activity, whereas *Pediococcus acidilactici* PAH and *Lactococcus lactis* ATTC 11454 showed no activity, which was also the case for *Carnobacterium gallinarum* LMG 9841 and *Carnobacterium divergens* LMG 9199. Four *C. maltaromaticum* strains (Cm 6-1, Cm 3-1, Cm 5-1, and ATCC 35586) showed weak activity, among which the Cm 3-1 and Cm 5-1 strains had delayed positive phenotypes during aerobic incubation at 25 °C (Table 1). The remaining strains, Cm 4-1 and Cm 1-2, gave negative results under these conditions. Incubation under microaerophilic conditions gave similar results.

An additional experiment examined citrate utilization of the two strains Cm 6-1 and ATCC 35586 during growth in APT broth with 22 or 30 mM citrate. Bacterial concentrations increased, irrespectively of initial citrate concentration, from log 6.1 CFU/mL (Cm 6-1) or log 6.3 CFU/mL (ATCC 35586) to log 8.7 CFU/mL (Cm 6-1) or log 9.1 CFU/mL (ATCC 35586) after 9 days of incubation at 25 °C. The medium with the lowest initial concentration of citrate (22 mM) contained either 18.7 (ATCC 35586; an average of three replica cultures) or 19.5 (Cm 6-1; an average of four replica cultures) mM citrate at end of incubation. One ATCC 35586 sample that only contained 12 mM was noted as an outlier and was not included in the average. Similarly, the medium with the highest initial concentration of citrate (30 mM) contained 28.25 mM at the end of incubation irrespective of strain (average of four replica cultures in each case, see Table S2 for details). Taking into consideration that the analysis had a relative standard deviation of 3%, the results indicate that the two strains did not—or only to a limited extent—utilize citrate when present in relatively high amounts. Production of short-chain fatty acids was also examined in this experiment. Both strains showed limited production of acetic acid: 1.8 mM or 1.2 mM for the 3Ba-II-6 strain and 2.5 or 1.5 mM for the ATCC 35586 strain in APT broth with 22 or 30 mM citrate, respectively. Production of formic acid depended on strain: 11.1 or 9.1 mM for the 3Ba-II-6 strain and 2.4 or 0.6 mM for the ATCC 35586 strain in APT broth with 22 or 30 mM citrate, respectively. These values were calculated by subtraction of concentrations found in sterile media: 5.5 mM or 7.6 mM acetic acid and 2.5 or 4.7 mM formic acid in APT broth with 22 or 30 mM citrate, respectively.

**Figure 3:**
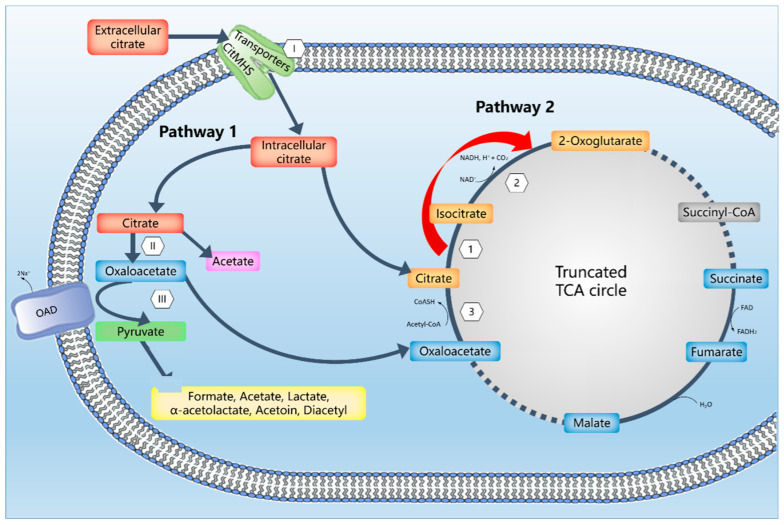
The prediction of citrate metabolism pathways in the *C. maltaromaticum* Cm 6-1 strain (see Table 1, Figure 1 and Figure 2 for a complete list of genes). Pathway 1 constitutes the typical citrate catabolism pathway with oxaloacetate as an intermediate. I: Citrate MHS transporter; II: citrate lyase complex; III: oxaloacetate decarboxylase complex. Pathway 2 includes isocitrate and α-ketoglutarate as intermediates. 1: Aconitate hydratase; 2: isocitrate dehydrogenase, 3: citrate synthase. A potentially associated citrate transporter is not shown in the figure.

Several genes, identified by genome annotations, were associated with two different pathways for citrate metabolism in *C. maltaromaticum* ( Figure 1, Figure 2 and Figure 3, Table S1). Pathway 1 constitutes the typical LAB citrate metabolism pathway and includes oxaloacetate as an intermediate, and pathway 2 includes TCA compounds as intermediates ( Figure 1, Figure 2 and Figure 3). All examined strains encoded the pathway 2 genes whereas only two strains encoded the pathway 1 genes (Table 1). A screening of whole-genome sequences of 14 additional *C. maltaromaticum* strains showed the presence of genes associated with pathway 1 in three other strains and the presence of genes associated with pathway 2 in all 14 strains (Figure S1). SNP analysis indicated that the *C. maltaromaticum* strains possessing pathway 1 genes are not necessarily closely related (Figure S1).

None of these pathways were detected in five strains of *Carnobacterium divergens* or one strain each for *Carnobacterium funditum* and *Carnobacterium viridans*, whereas pathway 1 was observed in one strain of *Carnobacterium pleistocenium.* The presence of pathway 1 genes in only some *C. maltaromaticum* strains indicate that their presence could either be due to a transfer event from another species or they have been lost from several lineages of *C. maltaromaticum*. In relation to the first possibility, we have previously discussed the capability of genome expansion in *C. maltaromaticum* compared to other *Carnobacterium* spp. [28]. BLAST analyses, as well as the alignment and construction of phylogenetic trees of predicted protein domains of *C. maltaromaticum* Cm 6-1 pathway 1 gene products, indicated that the most similar gene sequences were generally found in the related *Isobaculum melis*, with *Vagococcus salmoninarum* possessing the second-closest related set of genes (results not shown). It will be of interest to conduct further studies on whether citrate pathway 1 genes are localized on plasmids in order to evaluate the stability of their presence in individual strains.

The overall organization of the pathway 1 gene cluster is similar in the four strains Cm 4-1, Cm 6-1, JIP 2891, and 10040100629 (Figure 1 and Figure S2A). Following the *citM-CDEFGT* cluster nomenclature [29] and with the Cm 6-1 sequence as an example, the locus tag 3134 encodes a GntR family transcriptional regulator CitO that may regulate the activity of the *cit* promoters [30,31]. Locus tags 3135 and 3136 are hypothetical proteins probably associated with the active citrate lyase complex and similar to CitG and CitT [29]. Locus tag 3141 was predicted as a CitMHS transporter for citrate/H+ (CitM), a metal–citrate complex protein that transports the citrate^2-^–Mg^2+^ complex [29,32,33]. Locus tags 3142, 3143, and 3144 encoded CitDEF: citrate lyase alpha, beta, and gamma chains, respectively. Locus tag 3145 encoded CitC: citrate lyase ligase. The locus tags 3137 and 3140 encoded oxaloacetate decarboxylase (OAD) beta and alpha chains, respectively. 

In the *L. lactis* biovar. diacetylactis CRL264 strain, citrate transport, and metabolism are composed of the chromosomal *citM(ψcitO)-I-CDEFXG* cluster [34]. However, in the present study, we only observed the cluster of *citM(H)-CDEFGT(X)* without *citI* gene.

The gene products associated with locus tags 3138 and 3139 are related to transmembrane function; region and transmembrane helices were predicted for the 3139 gene product by use of the TMHMM server. These proteins may be associated with the functionality of the OAD-beta chain, and the latter was also predicted to contain transmembrane helices. Transmembrane OAD has previously been identified in LAB and has been associated with the type II citrate pathway in *E. faecalis* [12,35]. 

Soluble OAD are classified into three groups (EC 1.1.1.38, EC1.1.1.39, EC1.1.1.40) based on the malic enzymes (MEs). Notably, the soluble OAD (EC1.1.1.40) was identified in the Cm 6-1 strain (locus tag 1547), indicating an additional potential ability to convert oxaloacetate to pyruvate [36,37].

Pathway 2 includes the conversion of citrate into isocitrate and α-ketoglutarate by a truncated tricarboxylic acid (TCA) cycle containing the genes encoding aconitate hydratase and isocitrate dehydrogenase but lacking the genes encoding the reactions from α-ketoglutarate to succinate (Figure 2, Figure 3, and Figure S2B). This pathway is described for other LAB [38,39,40]. The pathway 2 genes clustered together with a gene encoding citrate synthase that synthesizes citrate from oxaloacetate and acetyl-CoA (Figure 2, Figure 3, and Figure S2B). Thus, oxaloacetate or citrate, perhaps imported by a presumptive transporter (L-malate or Citrate/H^+^ symporter, CimH, locus tag 1548 in Cm 6-1 sequence) encoded by a gene present in all six strains (Table 1) are presumable substrates.

The experimental conditions employed in the initial phenotypic screening of *Carnobacterium maltaromaticum* citrate degradation did not induce pathway 1, as indicated by the negative result for strain Cm 4-1 that contains the genes for pathway 1 but showed a negative phenotype (Table 1). Thus, the observed phenotype on citrate utilizing media appeared not to be associated with the presence of pathway 1 genes.

Furthermore, APT broth with 1% glucose and 22 or 30 mM citrate did not appear to support citrate utilization by *C. maltaromaticum* ATCC 35586 (with genes encoding pathway 2) or Cm 6-1 (with genes encoding pathways 1 and 2) or to affect the production of short-chain fatty acids to any significant extent. The presence of glucose may suppress the conversion of citrate in *E. faecalis* but not in *L. lactis*, *Lacticaseibacillus casei*, or *L. plantarum* [3,41,42]. As the medium employed in the initial phenotypical screening—which showed some citrate utilization as described above—also contains glucose, it is currently not clear what effect glucose might have on *C. maltaromaticum* citrate catabolism. Among other carbohydrates, galactose may exert an enhancing effect for citrate metabolism in *E. faecalis*, *L. casei*, and *L. plantarum*. Suppression of citrate metabolism by lactose was not observed in *L. casei*, *L. plantarum*, or *Leuconostoc mesenteroides* [3,43] and depended on the medium in *E. faecalis* [44]. 

pH also affects citrate catabolism, with the highest activity observed between pH 5.5 and 6.0 in *Lc. lactis* and *Leuconostoc* spp. [45], whereas other studies show that low pH values increased activity in *Lc. lactis*, *L. casei*, and *L. plantarum* [3,46]. Citrate may also be an inducer, as shown for *E. faecalis* and *L. mesenteroides* [31,47,48,49], although this compound does not appear as a satisfactory condition for *C. maltaromaticum* during growth in APT broth with citrate.

Succinate is a product of the reductive TCA cycle for some LAB, such as strains belonging to *L. pentosus* and *L. plantarum* [50,51]. Implicated enzymes include malate dehydrogenase, fumarase, and fumarate reductase [50,51]. Using RAST, we found a potential fumarate hydratase gene (=fumarase) (locus tag 2880 in the Cm 6-1 sequence) and a gene encoding a redox protein related to succinate dehydrogenase/fumarate reductase (locus tag 3513 in the Cm 6-1 sequence) in all genomes for strains included in Table 1, whereas there was no evidence for a malate dehydrogenase gene. This potential lack of one of the genes necessary for encoding such a pathway agrees with the lack of reports on the production of succinate by *C. maltaromaticum*. 

Pyruvate serves as a substrate for *C. maltaromaticum* for the production of lactic and acetic acid, formic acid, CO_2_, acetoin, diacetyl, and 2-3-butanediol [5]. Oxaloacetate and pyruvate may be converted into α-ketoglutarate by the aspartate aminotransferase and alanine aminotransferase enzymes, respectively [49]. These two enzymes are widely detected in various LABs. Isocitrate dehydrogenase in pathway 2 also converts isocitrate into α-ketoglutarate (Figure 3). The α-ketoglutarate compound may then undergo a transamination reaction with leucine, resulting in the formation of 3-methyl butanal and 3-methyl butanol. These two compounds are also produced by *C. maltaromaticum* [49,52,53,54]. The overall effects of citrate on pyruvate and α-ketoglutarate catabolism in *C. maltaromaticum* are not known. It will be of interest to examine this further, especially with regard to the synthesis of the flavor compounds diacetyl and 3-methyl-butanal.

In conclusion, this study shows that some strains of *C. maltaromaticum* appear to encode two different pathways for metabolizing citrate. To our knowledge, this is the first genetic analysis on potential citrate metabolism pathways for the *Carnobacterium* genus. Currently, the conditions that activate the citrate pathways are not known and further studies are required to illuminate them. As this species has potential as an adjunct culture in soft cheese products, it would also be of interest to investigate whether citrate catabolism in *C. maltaromaticum* by pathway 1 and/or pathway 2 may result in the production of flavor compounds under conditions prevailing in such products. We are currently researching that direction by examining the external regulation of citrate catabolism in *Carnobacterium maltaromaticum*.

## Figures and Tables

**Table 1:** Overview of phenotypical and bioinformatical analyses for selected strains of *Carnobacterium maltaromaticum*.

Strain	Isolated from	Citrate Metabolic Phenotype ^a^	Genes Encoding Specific Pathway		Ref.
			Pathway 1 ^b^	Pathway 2 ^c^	
Cm 4-1^d^	*Sphagnum* pond	−	+	+	[13]
Cm 6-1	*Sphagnum* pond	(+)	+	+	[13]
Cm 1-2	*Sphagnum* pond	−	−	+	[13]
Cm 3-1	*Sphagnum* pond	(+)(d)	−	+	[13]
Cm 5-1	*Sphagnum* pond	(+)(d)	−	+	[13]
ATCC35586	Diseased salmon	(+)	−	+	[6]

^a^ indicates a negative phenotype, (+) indicates a weak positive phenotype, (d) = delayed reaction. ^b^ See Figure 1B and Table S1 for a list of genes. ^c^ See Figure 2B and Table S1 for a list of genes. ^d^ Numbers for *Sphagnum* pond isolates as given in Leisner et al. [13]. The first number indicates in each case a specific rep-PCR cluster.

## Data Availability

The genome sequences of Cm 4-1, Cm 6-1, Cm 1-2, Cm 3-1, and Cm 5-1 strains (BioProject ID: PRJEB40278) and of JIP 2891 and 10040100629 strains (BioProject ID: PRJEB40429) were uploaded to GenBank. The *Carnobacterium divergens* Cd 1-1 sequence was from the RAST annotation server (job ID #692064). Sequences of ATCC 35586, LMA 28, and the remaining strains were downloaded from GenBank. Accession numbers are listed in Figure S1.

## Supplementary Materials

The following are available online at https://www.mdpi.com/article/10.3390/microorganisms9102169/s1, Table S1. Compilation of genes related to citrate metabolism in *Carnobacterium maltaromaticum* 3Ba-6-II. Table S2. The citrate utilization of the two *Carnobacterium maltaromaticum* strains Cm 6-1 and ATCC 5586. Figure S1. Comparison of SNP cladogram with the absence or presence of citrate pathway 1 and 2 genes in published genomes of *C. maltaromaticum* as well as other *Carnobacterium* spp. The filled squares indicate the confirmed presence while empty squares indicate the absence of pathway genes. The assembly accession includes IDs/numbers from NCBI bioproject, GenBank accession numbers, and RAST annotation server IDs. Figure S2. (A) Organization of pathway 1 gene cluster in the strains Cm 4-1, Cm 6-1, JIP 2891, and 10040100629. Mobile elements were situated next to the gene encoding the citrate lyase ligase in the 10040100629 strain. (**B**) Organization of truncated pathway 2 gene cluster in the nine strains Cm 5-1, Cm 3-1, Cm 6-1, Cm 1-2, Cm 4-1, JIP 2891, 10040100629, LMA28, and ATCC 35586.
