# Supplementary material for: Identification of Potential Citrate Metabolism Pathways in Carnobacterium maltaromaticum
Source: Microorganisms. 2021 Oct 18;9(10):2169. doi: 10.3390/microorganisms9102169 (PMC8537297; doi:10.3390/microorganisms9102169)
Supplement: Supplementary file 1 [file microorganisms-09-02169-s001.zip › microorganisms-1415841 suppl final OK.pdf]

Supplementary Materials

Table S1. Compilation of genes related to citrate metabolism in *Carnobacterium maltaromaticum* 3Ba-6-II.

Gene product	Pathway	Locus	Proposed Function
Hypothetical protein; transcriptional regulator, GntR family	1	3134	Regulator
Hypothetical protein; CitG; triphosphoribisyl-dephospho-CoA synthases	1	3135	Associated with active citrate lyase complex, similar to CitG
Hypothetical protein; CitT(CitX); an acyl carrier protein	1	3136	Associated with active citrate lyase complex, similar to CitT
Oxaloacetate decarboxylase beta chain/Methylmalonyl-CoA decarboxylase	1	3137	EC 4.1.1.3, now EC 7.2.4.2/(EC 4.1.1.41)
Hypothetical protein; biotin/lipoyl attachment domain	1	3138	Transmembrane function; associated with functionality of OAD beta chain
Hypothetical protein; transmembrane function	1	3139	Transmembrane function; associated with functionality of OAD beta chain
Oxaloacetate decarboxylase alpha chain	1	3140	EC 4.1.1.3
Citrate/H+ symporter of CitMHS family	1	3141	Citrate-iron uptake permease
Citrate lyase alpha chain	1	3142	EC 4.1.3.6
Citrate lyase beta chain	1	3143	EC 4.1.3.6
Citrate lyase gamma chain, acyl carrier protein	1	3144	EC 4.1.3.6
[Citrate [pro-3S]-lyase] ligase	1	3145	EC 6.2.1.22
Aconitate hydratase	2	2652	EC 4.2.1.3
Isocitrate dehydrogenase	2	2651	EC 1.1.1.42
Citrate synthase	2	2650	EC 2.3.3.1
Citrate synthase	2	2339	EC 2.3.3.1
Similar to citrate lyase beta subunit	?	403	E.C. 4.1.3.6
L-malate or citrate/H+ symporter CimH (TC 2.A.24.2.4)	2?	1548	Transporter
NADP-dependent malic enzyme soluble oxaloacetate	2?	1547	EC 1.1.1.40

Table S2. The citrate utilization of the two *Carnobacterium maltaromaticum* strains Cm 6-1 and ATCC 5586.

**Filename**	**MS-Omics ID**	**Customer ID**	**Citric acid (*mM*)**	**Average concentration**
210916-1-016-CS27370.cdf	CS27370	APT	22	22
210916-1-031-CS27371.cdf	CS27371	APT +	30	30
210916-1-026-CS30445.cdf	CS30445	ATCC 35586/+/1	27	28.25
210916-1-013-CS30446.cdf	CS30446	ATCC 35586/+/2	28	
210916-1-017-CS30447.cdf	CS30447	ATCC 35586/+/3	29	
210916-1-021-CS30448.cdf	CS30448	ATCC 35586/+/4	29	
210916-1-029-CS30449.cdf	CS30449	ATCC 35586/-/1	18	18.7
210916-1-015-CS30450.cdf	CS30450	ATCC 35586/-/2	19	
210916-1-027-CS30451.cdf	CS30451	ATCC 35586/-/3	19	
210916-1-012-CS30452.cdf	CS30452	ATCC 35586/-/4	12	Not included
210916-1-023-CS30453.cdf	CS30453	Cm 6-1/+/1	28	28.25
210916-1-028-CS30454.cdf	CS30454	Cm 6-1/+/2	28	
210916-1-019-CS30455.cdf	CS30455	Cm 6-1/+/3	28	
210916-1-014-CS30456.cdf	CS30456	Cm 6-1/+/4	29	
210916-1-022-CS30457.cdf	CS30457	Cm 6-1/-/1	18	19.5
210916-1-024-CS30458.cdf	CS30458	Cm 6-1/-/2	19	
210916-1-030-CS30459.cdf	CS30459	Cm 6-1/-/3	20	
210916-1-020-CS30460.cdf	CS30460	Cm 6-1/-/4	21	

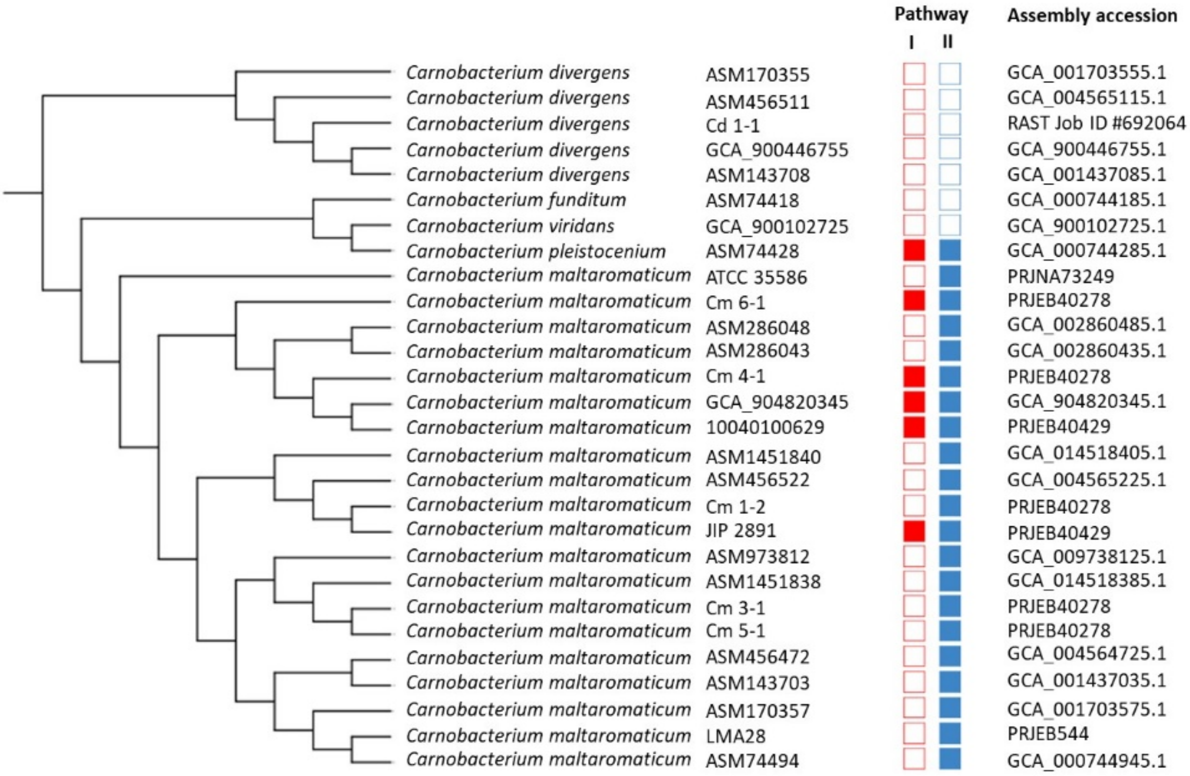

Figure S1. Comparison of SNP cladogram with the absence or presence of citrate pathway 1 and 2 genes in published genomes of *C. maltaromaticum* as well as other *Carnobacterium* spp. The filled squares indicate the confirmed presence while empty squares indicate the absence of pathway genes. The assembly accession includes IDs/numbers from NCBI bioproject, GenBank accession numbers, and RAST annotation server IDs.

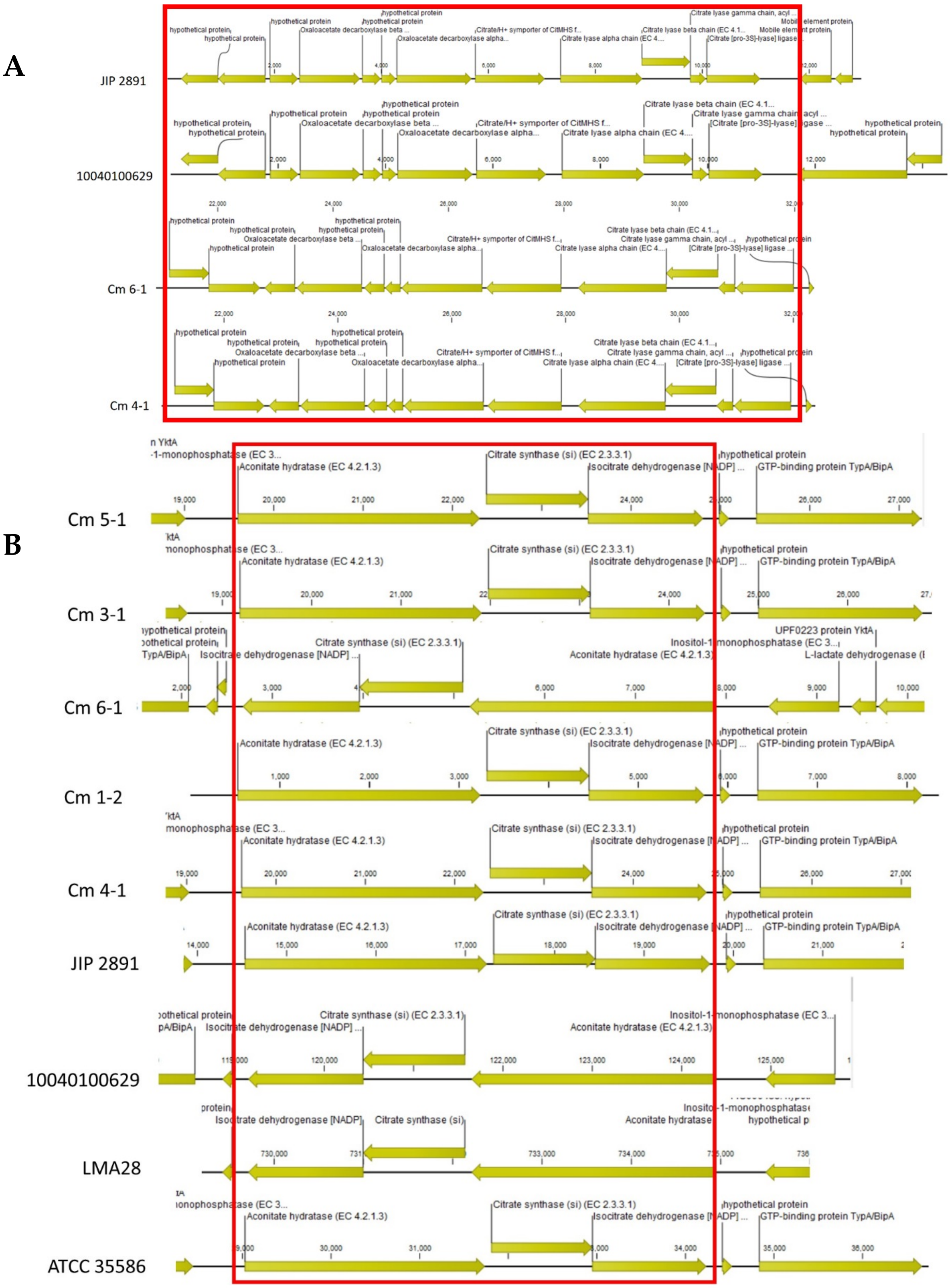

Figure S2. (**A**) Organization of pathway 1 gene cluster in the strains Cm 4-1, Cm 6-1, JIP 2891, and 10040100629. Mobile elements were situated next to the gene encoding the citrate lyase ligase in the 10040100629 strain. (**B**) Organization of truncated pathway 2 gene cluster in the nine strains Cm 5-1, Cm 3-1, Cm 6-1, Cm 1-2, Cm 4-1, JIP 2891, 10040100629, LMA28, and ATCC 35586.
